# New evidence of a Roman road in the Venice Lagoon (Italy) based on high resolution seafloor reconstruction

**DOI:** 10.1038/s41598-021-92939-w

**Published:** 2021-07-22

**Authors:** Fantina Madricardo, Maddalena Bassani, Giuseppe D’Acunto, Antonio Calandriello, Federica Foglini

**Affiliations:** 1 CNR-National Research Council, ISMAR-Marine Science Institute in Venice, Castello 2737/f, 30122 Venice, Italy; 2 Università Iuav di Venezia, Santa Croce 191 Tolentini, 30135 Venice, Italy; 3 CNR-National Research Council, ISMAR-Marine Science Institute in Bologna, Via Gobetti, 101, 40129 Bologna, Italy

**Keywords:** Solid Earth sciences, Geomorphology

## Abstract

This study provides new evidence of the presence of an ancient Roman road in correspondence to a paleobeach ridge now submerged in the Venice Lagoon (Italy). New high resolution underwater seafloor data shed new light on the significance of the Roman remains in the lagoon. The interpretation of the data through archive and geo-archaeological research allowed a three-dimensional architectural reconstruction of the Roman road. The presence of the ancient Roman road confirms the hypothesis of a stable system of Roman settlements in the Venice Lagoon. The study highlights the significance of this road in the broader context of the Roman transport system, demonstrating once more the Roman ability to adapt and to handle complex dynamic environments that were often radically different from today.

## Introduction

The Romans built a very efficient road system extending for tens of thousands of kilometres to connect all their territories^1–4^. Several portions of this ancient road network are still well preserved after more than two millennia in many archaeological sites in Europe, the Middle East and North Africa^5^. The transport system, however, was not limited to the routes on land, since the imperial control of the territory extended to transitional environments such as deltas, marshes, and lagoons^6–8^ and a capillary network of waterways was used for the exchanges of goods and the movement of people.

**Figure 1:**
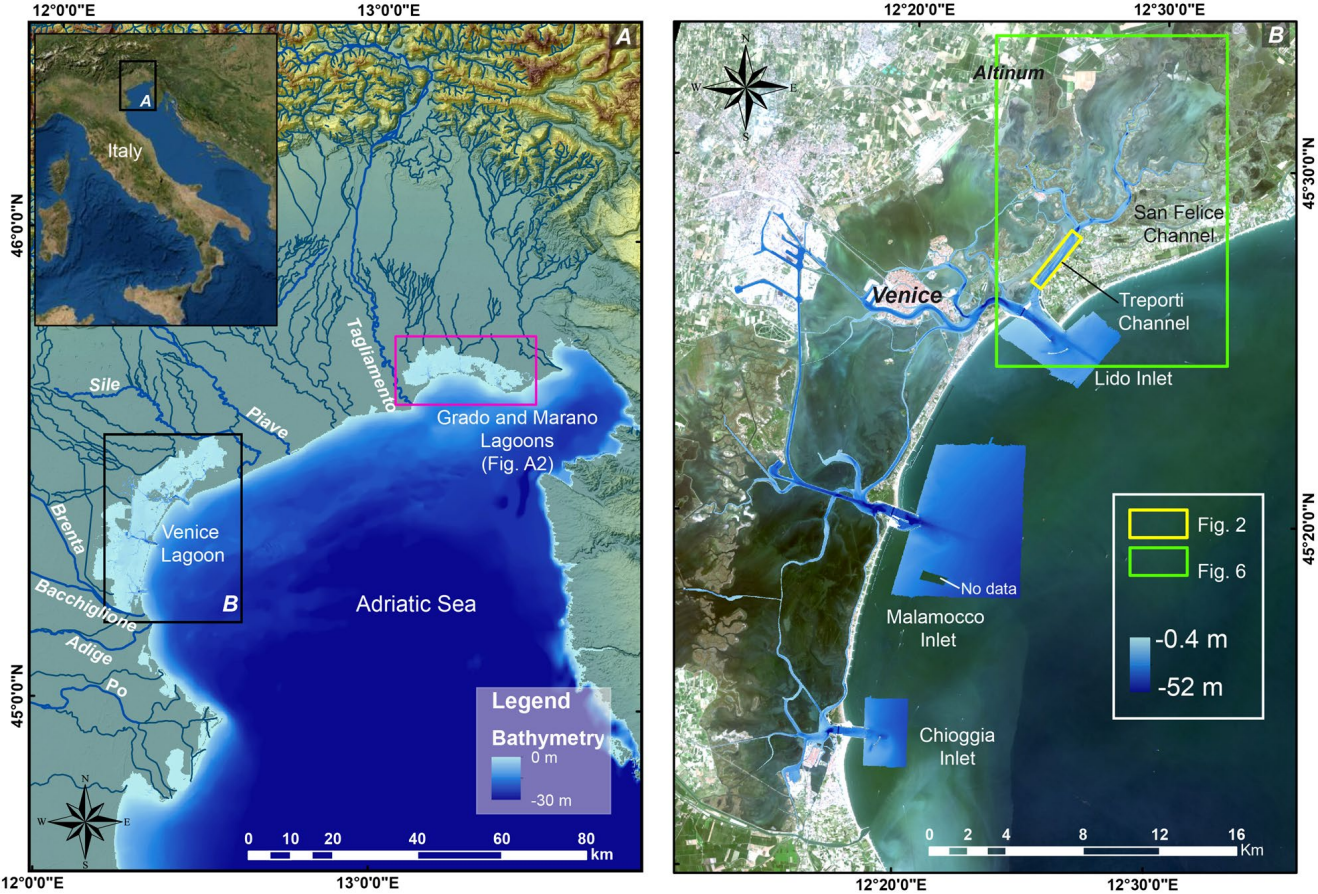
(**A**) The study geographical setting in the North-East of Italy with the location of the Grado and Marano Lagoon (in the pink box, Figure A2 in Supplementary Material); (**B**) The bathymetry of the Venice Lagoon tidal channels and inlets^120^ and the study area in the northern part of the Venice lagoon. The yellow and green boxes represent the location of Figs. 2 and 6, respectively. Satellite image source: Esri DigitalGlobe, GeoEye, i-cubed, USDA,USGS,AEX,Getmapping, Aerogrid, IGN, IGP, swisstopo, and the GIS User Community, https://services.arcgisonline.com/ArcGIS/rest/services/World_Imagery/MapServer.

In this context, what was the role played by the Venice Lagoon, the largest lagoon in the Mediterranean Sea, surrounding the historical city of Venice (Italy) (Fig. 1)? We know that in Roman Times, the relative mean sea level was lower than today and large parts of the lagoon, which are now submerged, were accessible by land^9–13^. The fate of the Venice Lagoon, its origin and geological evolution have always been tightly linked to the relative mean sea level rise, that is now threatening the existence itself of the historical city and the lagoon island^14,15^.

However, whereas the geological evolution of the Venice Lagoon has been investigated in several studies, less is known about the human presence in the lagoon before the foundation of the historical city. Similarly to what happened to many coastal areas in the Mediterranean area^16,17^ and in other parts of the world^18–21^, several archaeological remains were found underwater or even buried under the lagoon seafloor, many of which of Roman origin^11^. The significance of these Roman remains was the object of a long-running debate: starting from the pioneering works of Dorigo^22^ and Canal^10,11,23^, most studies show a diffuse occupation of the lagoon already in Roman Times^24–30^. Other studies, however, excluded the possibility of the existence of permanent structures and infrastructures before the V–VI century AD^31–33^: Venice was thought to be built in a ‘desert’ place without any previous traces of human presence and the Roman findings on and within the seafloor belonged to buildings in the mainland surrounding the lagoon.

Within this long-running debate, this multidisciplinary study supports the perspective of extensive Roman settlements in the Venice Lagoon, by presenting new high resolution multibeam echo-sounder data interpreted through the archive and geo-archaeological research and digital modelling. In particular, the paper aims to: (i) show the presence of an extended road feature in the shallowly submerged lagoonal context of Venice as an indication of the development of settlement, movement, and economy in the area during Roman Times; (ii) confirm the Roman ability to adapt and to handle complex dynamic environments that were often radically different from today and (iii) finally, stress the need to rediscover, document and preserve the archaeological remains in submerged or buried coastal landscape that may be threatened by human induced changes or relative mean sea level rise.

## Results

### The morpho-bathymetric reconstruction

**Figure 2:**
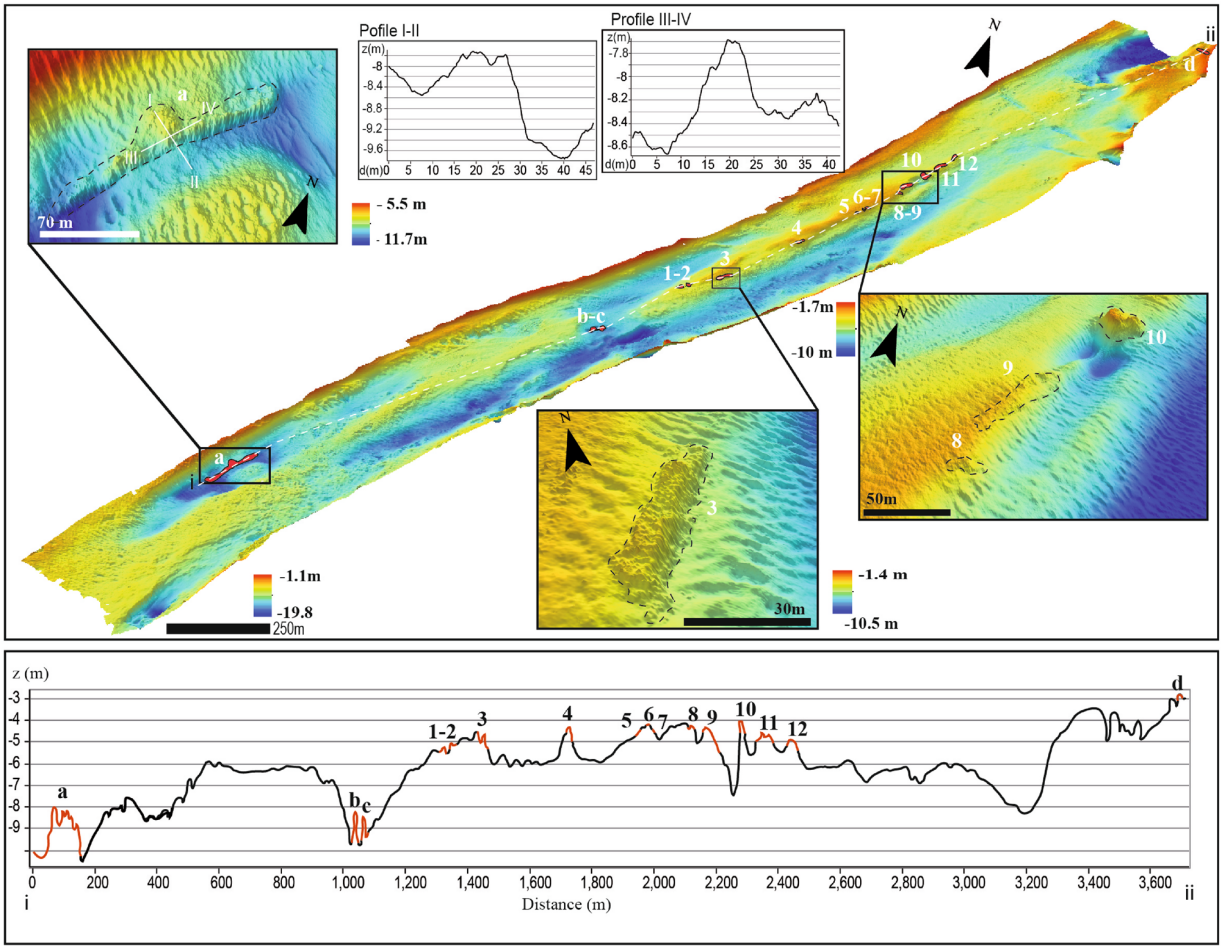
The Venice Lagoon and the bathymetry of the tidal channels. High resolution bathymetry of the Treporti Channel (DTM horizontal resolution 0.2 m, vertical exaggeration 5 x). The numbers 1 to 12 indicate the alignment of structures whose properties are summarized in Table 1. The letters a to d identify other structures found in the area. The zoom-in pictures show the detail of some of the archaeological structures: the sites 3, 8–9 and 10 (bottom-right) and the structure a (top-left), with the profiles I–II and III–IV, described in the text. The lower part of the picture represents the bathymetric profile extracted along all identified structures (white dashed line).

The Venice Lagoon is located at the northern end of the Adriatic Sea at the eastern edge of the Venetian coastal plain (Fig. 1). The lagoon is separated from the sea by narrow sandy beach ridges, aligned in a SW-NE direction interrupted by three tidal inlets. It has an average depth of less than 1 m. Within the lagoon there are intertidal and submerged mudflats, salt marshes, channels, creeks, and islands. This study focused on the Treporti Channel in the Northern Lagoon (Figs. 1 and 2). The bathymetry map of the Treporti Channel shows the presence of a submerged ridge along the SW-NE direction. Over the ridge between 4.3 to 5.3 m water depth, there are 12 different morphological reliefs (from 1 to 12) aligned for about 1140 m, parallel to the main channel axis oriented in the SW-NE direction (Fig. 2, Table 1). Given the regular shape of the reliefs, we interpret them as anthropogenic features, possibly archaeological structures. The length of the structures 1–8, 11 and 12 varies from about 5 to 38 m. Their width ranges from about 2 to 10 m. In the bottom-right part of Fig. 2, we show structures 3, 8–9 that were the object of archaeological investigation that we will discuss further on. Structure 3 has an almost rectangular shape with a steep south-east side, a width of 6 m and a length of 48 m. The structures 8 is of smaller dimensions (4.8 m × 14.3 m), whereas structure 9 is elongated with a width of 9 m and length of 52.7 m. They are less coherent and made of sparse small reliefs intermittently distributed on a large area, whereas structure 10 has a more circular shape.

**Table 1:** Dimensions and depth of the structures on the Treporti Channel seafloor shown in Fig. 2.

Structure number	Width (m)	Length (m)	Area (m^2^)	Perimeter (m)	Max depth (m)	Min depth (m)	Mean depth (m)	Max height (m)
1	6.2	18.8	89.1	43.4	5.7	5.0	5.3	0.7
2	3.8	15.8	46.8	34.5	5.5	4.8	5.2	0.7
3	6.0	48.0	214.6	102.5	5.7	4.2	4.9	1.5
4	5.3	21.9	87.3	48.2	4.8	4.0	4.4	0.8
5	4.1	13.6	35.7	29.4	4.7	4.2	4.4	0.5
6	2.2	5.2	8.3	12.5	4.5	4.1	4.3	0.4
7	4.0	11.6	32.2	25.9	4.7	4.2	4.5	0.5
8	4.8	14.3	49.1	32.4	5.4	4.2	4.6	1.3
9	9.0	52.7	316.2	109.7	6.3	4.0	4.9	2.3
10	27.0	31.7	451.5	114.2	6.3	3.6	4.9	2.7
11	10.6	38.2	302.1	84.5	5.5	4.3	4.8	1.2
12	6.8	36.8	196.6	79.8	5.5	4.7	5.1	0.8
a	22.4	134.8	706.6	256.0	11.4	7.4	9.0	4.0
b	11.6	21.9	107.0	56.6	9.8	7.6	8.7	2.2
c	7.5	14.5	79.6	37.1	10.3	7.9	9.3	2.4
d	8.6	47.4	248.4	100.3	3.6	2.2	2.8	1.4

Another group of structures, a, b, c in Fig. 2, lay deeper, at an average depth ranging between 8.7 and 9.3 m (Table 1), whereas the structure d is much shallower, with average depth of 2.8 m. The structure labelled by a (in the inset to the top left of Fig. 2), is the largest identified in the Treporti Channel occupying an area of more than 700 m$^2$ with a total length of about 135 m and a maximum width of about 22 m. In the center, it has an almost circular structure with a section of about 15 m and 10–12 m in the perpendicular direction (profiles I–II and III–IV of Fig. 2).

### The stratigraphical evidences

**Figure 3:**
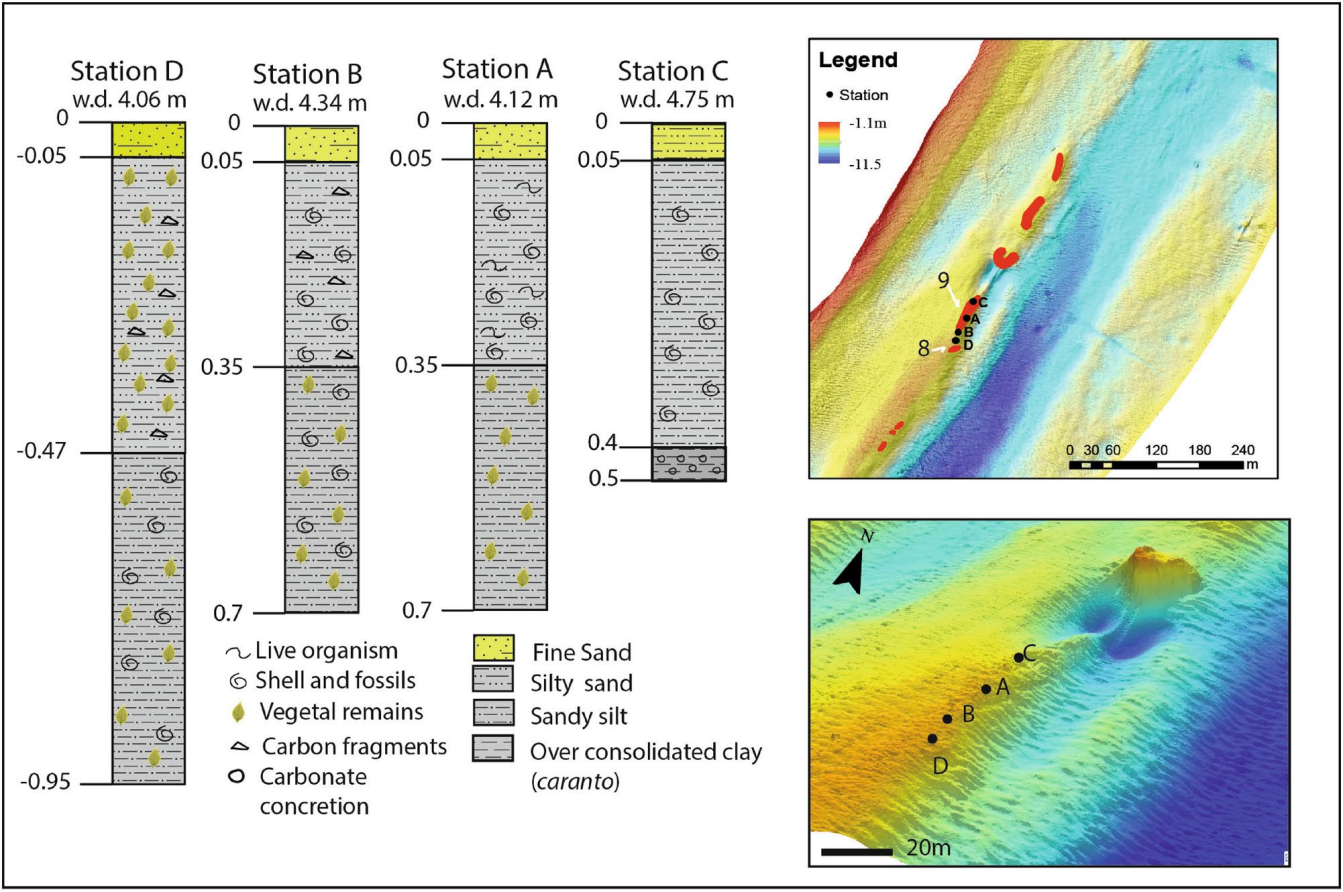
Stratigraphy of cores extracted below the archaeological layer under the structures 8 and 9 (Fig. 2) and their position on the bathymetric map.

Four cores were extracted in 1985 under the structures 8–9 (Fig. 3) during and extended archaeological investigation (Fondo Nausicaa n. 1902). With a length varying from 0.5 to 0.9 m under the archaeological layer, all cores presented lagoonal sediments overlaid by an upper fine sand layer and microfauna typical of a littoral environment (Cores A–D, Fig. 3). The cores and the microfauna showed the transition from a shallow lagoon environment to a clearly littoral place due to the backing of the coastline.

At a depth of about 5 m, the core C highlighted the presence of a layer of over consolidated clay with abundant carbonate concretions that was interpreted as the locally called “caranto” paleosol, lying under the lagoonal sediments. This paleosol formed by weathering as a result of sub-aerial exposure of the Pleistocene paleo-plain. It represents the last continental Pleistocene deposition marking the transition to the marine-lagoonal Holocene sediments^34,35^. The depth of this boundary varies within the lagoon area: it is more superficial at the inner margin of the lagoon deepening to the southeast, towards the Adriatic Sea^36–38^. This paleosol is not continuous and has a non-uniform pattern, with its ridges and dips having a fluvial origin^38,39^.

### The archaeological evidence

**Figure 4:**
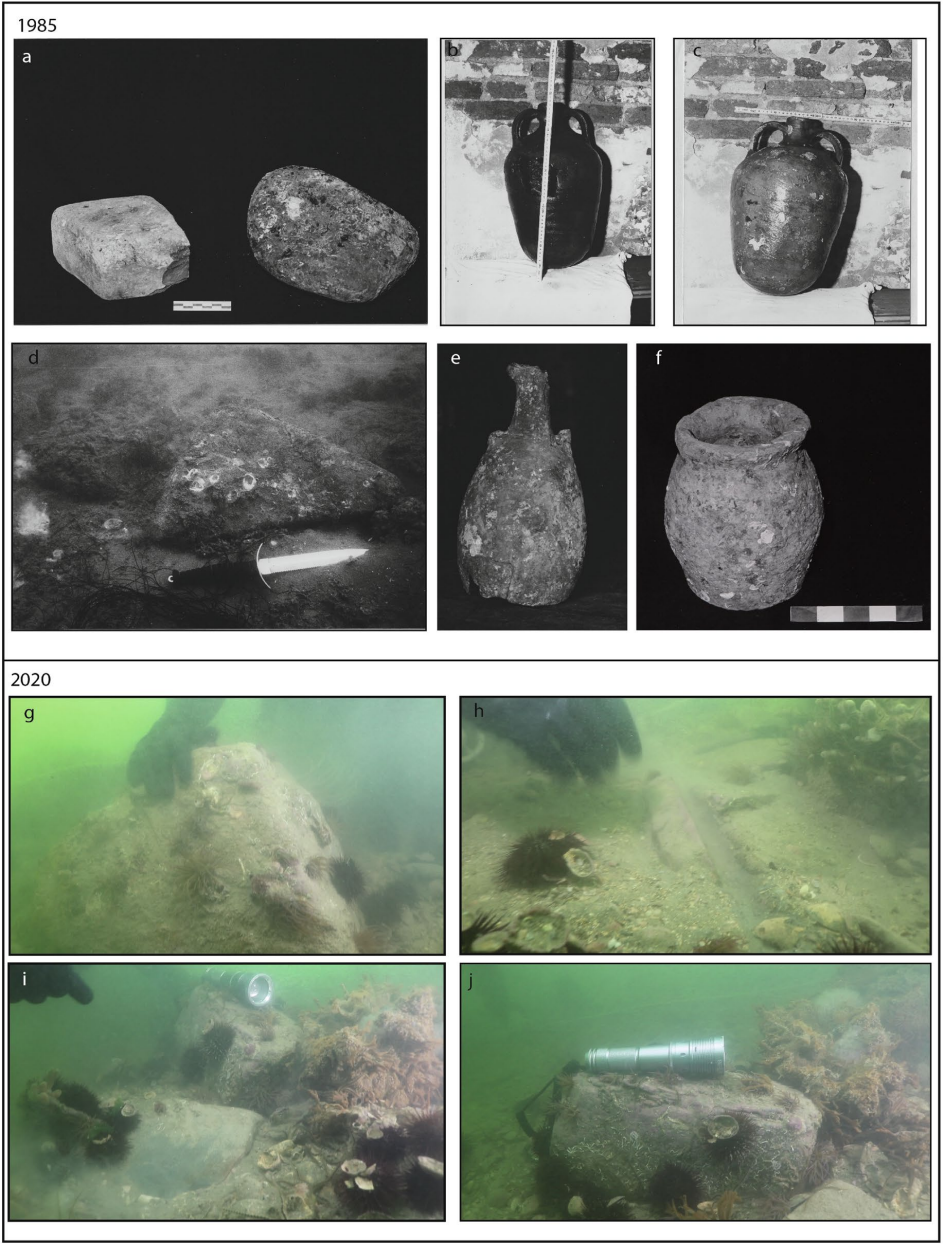
*Upper part*. Pictures of material discovered in the Treporti Channel in correspondence to the structure 8–9 (Figs. 2, 3) in 1985: (**a**) *Basoli*; (**b**,**c**) small amphora; (**d**) *basoli* on the seafloor; (**e**) Dressel 6A type amphora; (**f**) vase discovered in 1985; *Lower part*. Pictures extracted from the videos recorded by the divers of the *Nucleo Sommozzatori della Polizia di Stato* during the shooting of a documentary (see Supplementary Material) in different points along the structure 3, depicted in Fig. 3: (**g**) about 50 cm × 50 cm cubic stone found close to the flat stones; (**h**) preserved alignment of flat stones (*basoli?*); (**i**) inclined flat stone (*basolo?*) flanked by square stones in alignment; (**j**) detail of the square stone flanking the (*basolo?*).

The area was investigated in 1985 and in 2020 in correspondence to the sites 8–9 and 3, respectively (Figs. 2 and 3). As described in the report dated in 1986 (Fondo Nausicaa n. 1902), the first survey found a structure of an estimated length of 70 m, with a width varying from 5 to 7 m at an average depth ranging between 4 to 6 m. The structure was characterized by multiple lytic elements, which seemed to indicate, by morphology and disposition, the shape of an ancient road. The observed stones had an upper smooth face, whereas the lower part had an ovoidal shape (Fig. 4a,d). This lithic morphology was similar to the one of the Roman *basoli*, stones normally used to cover the upper surface of ancient roads. The *basoli* were disposed in parallel to the channel axis, as if they were following the SW-NE direction. Some of them are now preserved in the storage of the ‘Soprintendenza Archeologia, belle arti e paesaggio’ of Venice (Isola del Lazzaretto Nuovo, management: Gerolamo Fazzini); their dimensions are about 38 cm × 27 cm × 13 cm. The petrographic analysis of a thin section carried out by Lorenzo Lazzarini showed that the stones were in flyshoidal sandstone. During the diving inspection carried out in the 1980s, three clay vases were recovered: a small amphora with double handles (h about 45 cm: Fig. 4b,c), in which some traces of oily material were found (maybe rapeseed oil); a big amphora (h about 70 cm: Fig. 4e) without handles lacking the bottom that seems an example of the Dressel 6A type, dated at the I cent. BC–I cent. AD; a third small vase (h about 36 cm) of rough workmanship (Fig. 4f). The second and the third vases may date back to the late Antiquity-early Medieval age.

The second investigation, carried out by divers in July 2020 during the shooting of a documentary (see Supplementary Material), focused on the structure 3 of Fig. 2. The divers collected seven videos along a transect parallel to the main axis of the structure 3. Analogously to what was found in 1985 for the structures 8 and 9, also in this case the videos collected by the divers showed the presence of numerous stone blocks: large squared blocks sparsely distributed (Fig. 4g) and stones in alignment laid flat on the bottom with compatible dimension to the standard roman *basoli* (Fig. 4h). In some part of the alignment, the flat blocks were laterally framed by larger stones (Fig. 4i,j).

## Discussion

To interpret the dataset in a broader perspective, we first consider the geomorphological and archaeological background of the study area. Then, we compare the archaeological artifacts with similar examples elsewhere, reconsidering all the elements in a wider historical horizon.

### Geomorphological background: the coastline position over the centuries

The geomorphological evolution of the Venetian coastal plain is summarized in^40^ and a general description of the geological and stratigraphic setting of the Venice Lagoon is provided by the Sheet 128 “Venezia”^41^ and the Sheet 148–149 “Chioggia-Malamocco”^42^ of the new Geological Map of Italy at the 1:50,000 scale (Carta Geologica d’Italia alla scala 1:50,000). The origin and evolution of Venice Lagoon is controlled by variations in relative sea level (RSL), related to the combined effects of eustasy, glacio-isostacy, and subsidence^12,43–45^.

The origin of the lagoon is set around 6000–7000 yrs BP^46^ as a consequence of the Holocene marine transgression. According to RSL curve reconstruction by Vacchi et al.^47^, the RSL in the area of the Venice and the Grado-Marano Lagoons was at $-9.3\pm 0.8$ m at $\sim 7.5$ ka BP rising to $-5.5 \pm 0.8$ m at $\sim 6.6$ ka BP and to about $\sim -3$ m at $\sim 5.5$ ka BP. During this transgression, the sea gradually flooded the alluvial palaeo-plain that occupied the northern epicontinental Adriatic shelf and several barrier-lagoon systems formed in progressively more inland positions^48–51^. According to the reconstruction based on the deep borehole VE 1 of about 950 m, drilled in Venice in 1971, a sedimentary record of Pleistocene and Holocene sand, silt, clay and peat underlay the lagoon^52,53^. Within this succession, the ‘caranto’, the last continental Pleistocene deposition, is a few decimeters to a few meters thick altered layer, which marks the transition to the marine-lagoonal Holocene sedimentation^34,35^. Above this layer there is the Holocene succession. This succession was described with very high spatial resolution thanks to seismic data, sedimentological, radiometric and micropalaeontological analyses in the northern^54–56^, central^57–59^ and southern lagoon^60–64^. These data allowed the reconstruction of the main ancient geomorphological features such as paleoriver beds, ancient tidal channels, ancient salt marshes and paleobeach ridges^54,56,58,59,61–63^.

The reconstruction of the Holocene succession identified different phases in the lagoon formation: (1) a first transgressive phase (from about 10,000 to 6500 B.P., with the end of the transgression set about 6000 yr B.P.), when a full back barrier depositional environment had formed; (2) a RSL stabilization phase characterized by an increase of sediment supply to the coast leading to deltaic and shallow-marine progradation that reached its maximum near Roman times^64^; (3) a second transgressive phase, in some sense accelerated by the human intervention, a sort of “human induced transgression”, that lasted until present^63,64^. Starting from the XIV century AD, major rivers (e.g. the rivers Bacchiglione, Brenta, Piave and Sile—Fig. 2) that were flowing into the lagoon were diverted to the north and to the south of the lagoon to avoid its silting up. In the last two centuries, other human modifications such as the construction of new jetties at the inlets, dredging of navigation channels and the more recent modifications on the inlets took place^65–67^.

**Figure 5:**
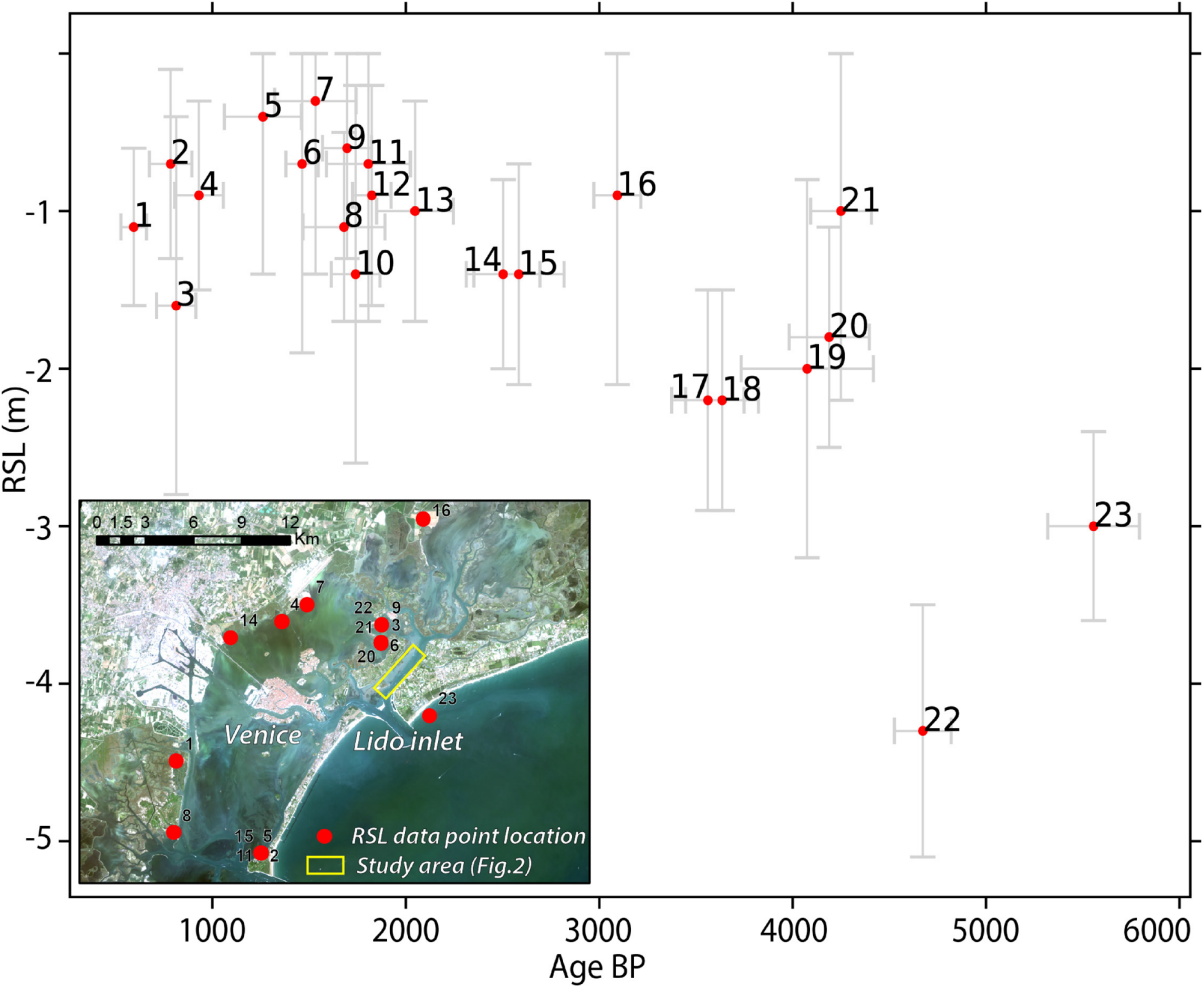
RSL data points (red dots) extracted from the database by Vacchi et al.^47^ and their location in the Venice Lagoon plotted as calibrated age against sea level relative to the present. The error bars represent the elevation and age errors.

In particular, the position of the coastline has changed over time. In the southern lagoon, different studies documented the presence of a paleobeach ridge indicating that the coastline had a more inland position during Roman Times^37,39,61,63^. In the central-southern lagoon, the reconstruction of Zecchin et al.^64^ and Donnici et al.^59^ suggest that the coastline position did not change much since Roman Times until present as it was hypothesized also by Bonardi et al.^68^. In the northern lagoon, instead, the coastline position varied over time in different climatic conditions showing inland migration during warm periods and seaward migration during climatic crises, respectively^40,68^. However, as observed in Vacchi et al.^47^, it is difficult to reconstruct the exact curve of the RSL in the late-Holocene: most likely due to compaction of the sediments, there is considerable scatter of the RSL index points (Fig. 5) for the area of the Venice Lagoon. These RSL points refer to internal areas of the Venice Lagoon, that could have had a rather different evolution of the area considered by the present work (Figs. 1, 2). This area underwent strong modifications over the last two millennia, making it hard to understand which could have been the local trend of RSL over time.

**Figure 6:**
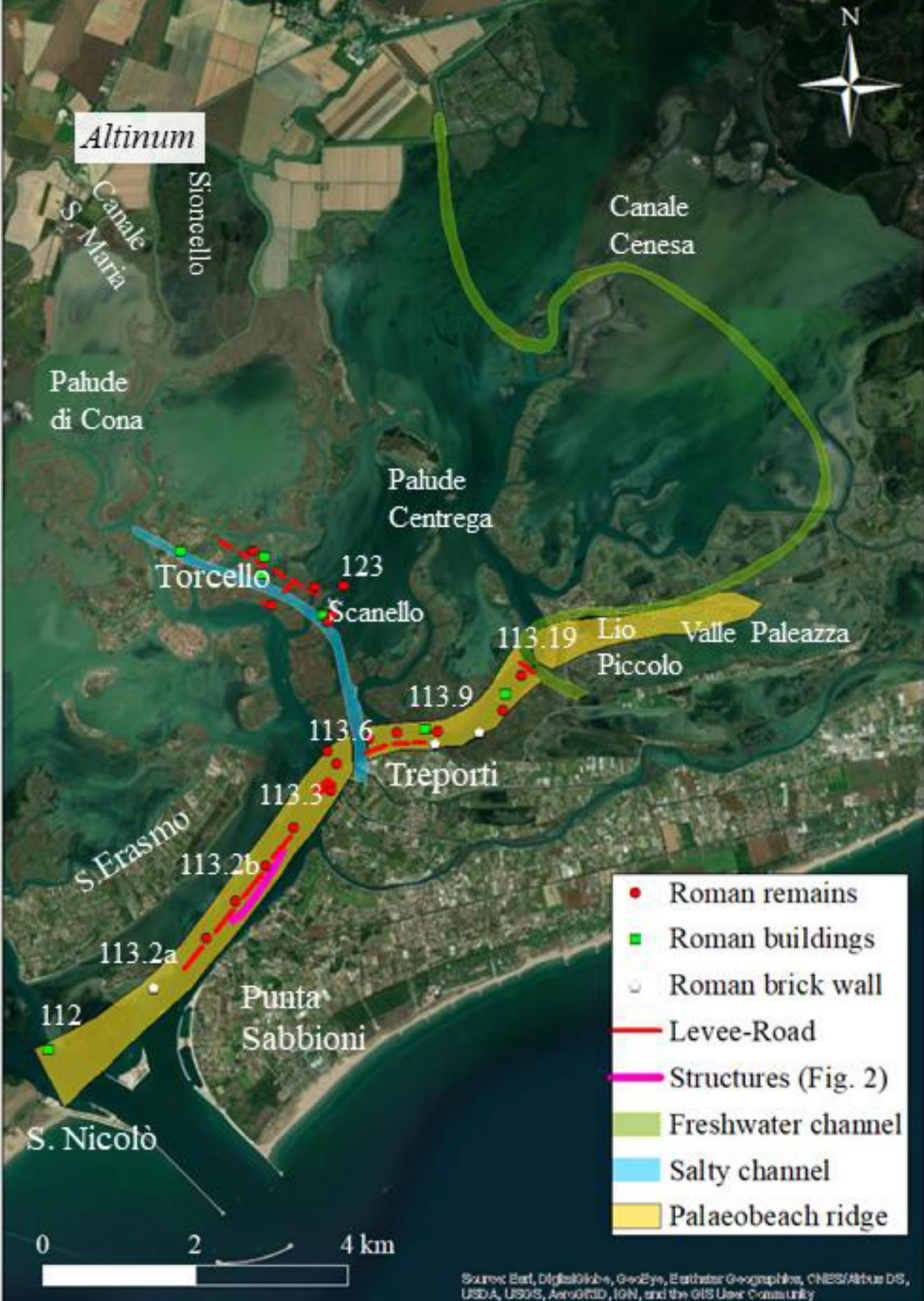
The position of the paleobeach ridge in the Treporti Channel in Roman Times (in yellow in transparency over the current satellite data) and the alignment of Roman lithic remains and levee road (red dots and lines), buildings (green squares) and brick walls (white pentagons); the map was modified from the Archaeological Map of the Venice Lagoon^11^. The pink solid line indicates the position of the structures reconstructed by this study (Fig. 2). Satellite image source: Esri DigitalGlobe, GeoEye, i-cubed, USDA,USGS,AEX,Getmapping, Aerogrid, IGN, IGP, swisstopo, and the GIS User Community, https://services.arcgisonline.com/ArcGIS/rest/services/World_Imagery/MapServer.

According to the geomorphological reconstruction of Bonardi et al.^40,68^, a paleobeach ridge occupied the present Treporti Channel in this area (Fig. 6) during Roman Times (2100–1800 yr B.P.), at the end of the RSL stabilisation. On this paleobeach ridge, Bonardi et al.^68^ for the first time referred to a submerged and partially buried alignment of lithic material stretching for about 3 km along the channel. Some of these structures were interpreted as part of Roman defense and observation buildings. The paleobeach ridge was successively submerged by mean sea level rise and partially destroyed. The coastline then shifted back to the S. Erasmo island position in the second transgressive phase^11,23,68^. The S. Erasmo island represented the littoral strip with two different inlets to the south and to the north for many centuries, as it is also documented by historical maps^69,70^.

The current configuration of the inlet, with the formation of the Treporti Channel and the new littoral is very recent. This configuration is a consequence of the diversion of the Piave River to the north of the lagoon and of the construction of the Lido inlet jetties between 1882 and 1910^69,71^. The sediments supplied by the Piave river and the presence of the jetties caused the deposition of large quantities of material to the north of the Lido inlet. In less than a few centuries, a new beach ridge (Treporti-Punta Sabbioni) and the Treporti Channel formed, reaching the present geomorphological configuration. This process is well documented by the direct quantitative comparison of the georeferenced historical bathymetry maps since 1809 to 2003^69,71^: Balletti et al.^69^ showed that since 1809 to 2003 the Treporti Channel underwent a strong erosion process becoming much deeper (from 2 to 8 m), whereas Fontolan et al.^71^ estimated that the Punta Sabbioni beach has accreted at a rate of more than 10 m/yr. since 1886 to 2003.

Finally, the area probably experienced a strong subsidence rate with respect to the internal lagoon and the city of Venice, due to natural and anthropogenic causes^39,65,72,73^. The shallow Holocene deposits are heterogeneous within the lagoon generating different land movements: the presence of thicker unconsolidated deposits generally corresponds to the largest subsidence rates. In a recent study based on satellite SAR interferometric methods, Tosi et al.^74^ observed an average land displacement up to 25 mm/year in correspondence with the newly built structures for the mobile barriers (MoSE) at the Lido inlet. The historical city of Venice, instead, experienced a relative sea level rise equal to 26 cm related to land subsidence and climate change effects over the last century^14^. Currently, however, the city and the central lagoon seem to be relatively stable with an average land subsidence of 1–2 mm/year^74^.

### Archaeological background: the Roman remains in the Venice Lagoon

Since the XVIII–XIX century AD, many scholars, historians and archaeologists have considered the Roman remains found in the Lagoon islands and channels as *spolia* from the Roman Venetian cities overlooking the lagoon. These *spolia* would have then been used for new buildings and decorative redeployments during the Medieval age and Renaissance. Mainly two factors contributed to the opinion that no Roman settlements could have been present in the Venice Lagoon before the V–VI century AD. First, the lagoon geomorphology changed greatly over the centuries. It was difficult to imagine an ancient landscape so different from the current one. The lagoon was seen as a separate space from the mainland, with few relations with the suburban areas of *Patavium* and *Altinum*, the Roman cities overlooking the Venice Lagoon. Secondly, only a minimal part of the buried and submerged findings and excavations made from the 1990s up to the beginning of the 2000s have been published.

The major ‘supporters’ of ancient settlements in the lagoon have been Ernesto Canal and Wladimiro Dorigo. During the 1980–1990s, thanks to surveys in the lagoon and archival-archaeological research^11,22,23,75,76^, they tried to demonstrate the existence of several Roman (and Pre-Roman) settlements in the Venice Lagoon. In their view, archaeological layers and ancient artefacts represented the traces of an extensive occupation of the lagoon until the birth of Venice in VIII–IX century AD. At the same time, Braccesi^77^ proposed new interpretations of ancient literary sources strengthening the hypothesis of an inhabited lagoon since Ancient Times.

The territory of *Altinum* (Figs. 1 and 6) probably extended with its harbour elements into the contemporary lagoon^78–81^. Some buildings in the modern Palude di Cona and Palude Centrega were interpreted as harbour structures and infrastructures of the ancient Roman city of *Altinum* (Fig. 6, 123^11^) or as residential buildings^82^. These structures may have been connected by levees-walkways now submerged and excavated in some places of the lagoon^83^. Furthermore, the archaeological diggings realized in Torcello^84–87^, with some new data also from archival research^26^, testified to the presence of Roman levels under the Medieval layers. This presence has been interpreted as proof of the existence of a private settlement during the imperial phase (a Roman villa?^26,30^), abandoned during the late Antiquity; then, a new phase of settlement occurred in the VI–VII century AD^88^, when the first Christian church was built. Important data also emerged from the studies and several stratigraphic diggings on Medieval sites in the lagoon^89–91^. The theory of a lagoon with Roman diffuse settlements, however, has still been opposed even recently^92^.

### The reconstructive hypothesis

The geomorphological studies reconstructed a different shoreline position during the Roman age (Fig. 6), with respect to the positions in pre-Roman and the Medieval phases. Here, the long coastal seaside could have been frequented at least in the Roman phase. The bathymetry data confirms the presence of a paleobeach ridge in the Treporti Channel (from now on, TC paleobeach ridge; Fig. 2). The alignment of the artifacts (structures 1–12, Fig. 2) extends on this paleobeach ridge, with the structures having about 4/6 m of depth, in agreement with what was found by the archaeological investigation in 1985 and by what was written by Canal^11^. The artifacts, however, lay below the available RSL values in the Venice Lagoon (Fig. 5), that vary between $-1 \pm 0.7$ m (point 13), $-1.4 \pm 1.2$ m (point 10) and $-0.7 \pm 0.7$ m (point 11) in Roman Times (about 2100–1800 years BP). As discussed before, this could possibly be related to the strong changes in terms of erosion and subsidence that occurred in the area.

**Figure 7:**
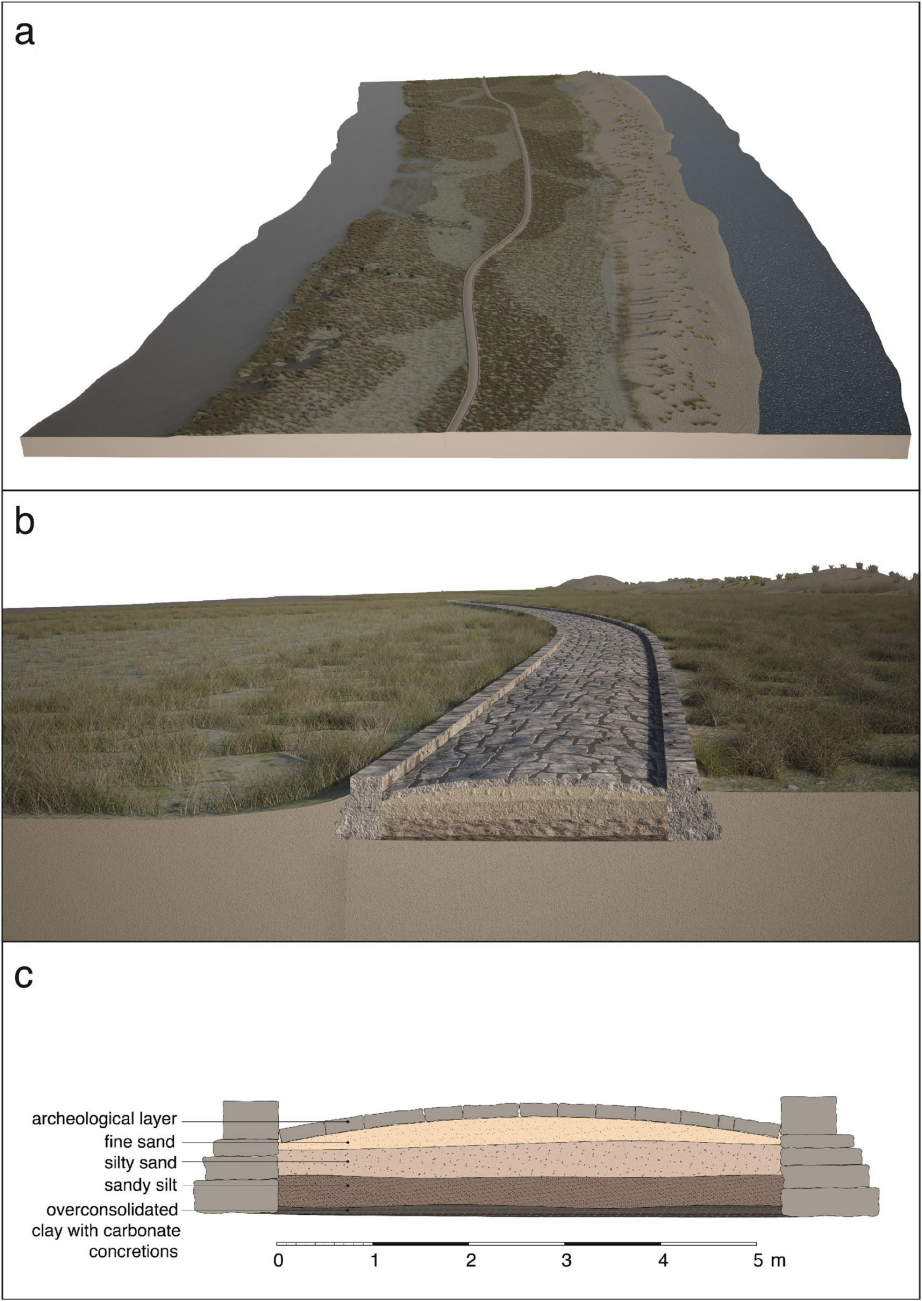
Reconstruction of the Treporti Channel paleobeach ridge and the Treporti Channel road (TC road)in Roman Times: (**a**) from an aerial perspective, with the Venice lagoon to the left and the Adriatic Sea to the right. The position of the TC road corresponds exactly to the position of the archaeological structures mapped (Fig 2), whereas the extension of the TC paleobeach ridge is only hypothetical since the area has been radically modified over the centuries; (**b**) a zoom-in view and (**c**) section of the TC road based on the stratigraphy of the cores extracted under the *basoli* (Fig. 3).

In his book, Canal described two main sites in this area, labelled with the codes 113.2b and 113.3 (see Fig. 7 for the location). Canal found that the site 113.2b was about 70 m long corresponding to the sites 8 and 9 of Fig. 2, whereas the site 113.3 was a very extended area (300 m) characterized, again, by a straight alignment of stone blocks arranged similarly to an ancient Roman road. Several fragments of roof tiles (*embrici*), sesquipedalian bricks, amphorae and other Roman vases were found around it). The diving inspection carried out in 2020 highlighted the presence of aligned stones also in correspondence to structure 3 (Figs. 2 and 4).

The discovery of the submerged structures of Fig. 2 confirms and further extends what was found by the archaeological investigation in 1985, described also by Canal^11^. In its current configuration, the alignment of the discovered artifacts extends for more than a thousand meters (1140 m) along the SW-NE direction with a width ranging from 2 to 10 m. They can be interpreted as residues of an ancient road, from now on called the Treporti Channel road (TC road), recognizable by the typical blocks of smooth stone on the surface, in this case sandstone, and the wedge-shape in their lower part.

The digital reconstruction illustrates this interpretative hypothesis (Fig. 7a): the TC paleobeach ridge is drawn between the Adriatic Sea (to the right) and the ancient lagoon (to the left) offering an overview of the ancient TC road. The digital and interactive system is the final step of a long procedural process starting from the reading and the analysis of the multibeam data to obtain a philological reconstruction of the lost ancient reality. Thus, the rendering of the TC road corresponds exactly to the position of the archaeological structures shown in Fig. 2. The extension of the TC paleobeach ridge, instead, can only be hypothesized: strong geomorphological changes occurred in the area in the last two millennia and the current bathymetry probably shows only what remains of the TC paleobeach ridge submerged and partially eroded over the centuries.

The reconstruction of the TC paleobeach ridge includes also elements of vegetation (Fig. 7a,b). Vegetation remains were found in the cores extracted under the structures 8–9 of the TC road (Figs. 2 and 3). Furthermore, traces of vegetation were recorded at 8–10 m in different other sites (Fig. 6, 113.6), as well as remains of minor paths: the traces found could suggest that along the coast it was possible to go through a land with luxuriant vegetation, maybe used for agricultural purposes too. The presence of vegetation has been recently confirmed and expanded in a study dedicated to the reconstruction of the plant landscape of the Venice Lagoon thanks to archaeobotanical findings^93^.

### The setting of the TC road in the Venice Lagoon

To the north-east of TC paleobeach ridge, an ancient building, possibly a defensive tower, was found (Fig. 6, 113.9). The building (dimensions about 8 × 8 m) was paved with sesquipedalian bricks, around which some Roman embrices, amphoras, glass and pottery fragments were found^11,24,25^. In the south-west part of TC paleobeach ridge, another similar ancient submerged defensive tower was identified (Fig. 6, 112). It had a square plan and was built with square stone blocks. As pointed out by Canal^11^, the TC road could have connected these two far-ends over the TC paleobeach ridge (Fig. 2, 113.2b) that separated the Adriatic Sea to the lagoon.

The presence of the TC road was probably related to the presence of waterways inside the lagoon. The paths of two natural ancient channels reconstructed by Canal are pictured in Fig. 6: a freshwater channel collected the waters of the rivers Sile-Piave from the land to the sea (Canale Cenesa in green in Fig. 6) and a salty channel extended from Torcello to Treporti (in light blue in Fig. 6^11^). The latter possibly represented the favourite route for transports from the Adriatic Sea to the *Altinum* harbour (and vice versa). The harbour, as we anticipated above, was probably located south of *Altinum*, close to the Scanello Channel, between the Palude di Cona and the Palude Centrega (Fig. 6) where multiple archaeological traces were found^11,23,25^. They seem to belong to big buildings, among which two possible warehouses (47 m × 42 m and 50 m × 46 m) with columns (base 2 m), structures for the port activities. The goods could have then reached the city thanks to the artificial channel called the ‘Sioncello’, realized during the I century BC: it was an inland waterway with mooring quay, that unified the Sile river (at that time a Piave branch) with the S. Maria channel^78,94^. Also, in the Scanello Channel, Canal^11^ identified the remains of what he considered a Roman road (Fig. 6, 123).

This would imply that the TC road was not an isolated infrastructure, but it was linked to other structures and infrastructures in the lagoon that served the circuit of the goods to and from *Altinum*. Indeed, other archaeological excavations in this area discovered remains of numerous levees-walkways now submerged^83^. Canal refers also to the presence of a levee-walkaway in the S. Felice Channel at NW of the TC road (Fig. 6, 113.19), that is documented also by Fozzati and Toniolo^83^. It was 12 m long at 6/8 m of depth and it was made of a wooden caging, amphorae, infill materials and stone blocks to cover the top of the way (depicted in Figure A1 in the Supplementary Material). In some points, amphorae Dressel 6A type, Dressel 7/11 and ‘north italic amphorae’ types dated at the I–II century AD constituted the base of the road. Fragments of plaster, a mortar, a dice cup, and other vases/amphorae dated at late Antiquity were found too. The amphorae of Dressel 6A type found in the Treporti Channel (Fig. 4e) can be compared with the ones used in the San Felice Channel for the base of the levee-walkway. The presence of late vases in both cases could be considered, in future detailed studies, as a marker of a restoration of the paths, as proposed by Fozzati and Toniolo^83^.

However, no traces of a wooden caging were found for the TC road. The presence of the “caranto” paleosol found in one of the cores under the TC road (station C, Fig. 3) could explain why: the TC road was probably built on a morphologically high (supratidal) hard substrate. The Pleistocene-Holocene boundary was higher in this area, as it was first shown by the comparative study of 18 cores extracted in the 1970s along the littoral strips^36,95^. In station C (Fig. 3), the “caranto” was found at a depth of about 5 m, i.e., at a much shallower position in comparison with other locations close to the littoral (see for example cores described in Rizzetto et al^37^, and Donnici et al^35^). This is confirmed also by the reconstruction of the Pleistocene-Holocene boundary depth in the area by Brambati et al^39^. The “caranto” palaeosol deposits are consolidated and much harder and resistant to deformation^35^. The *basoli* would have been then laid directly on the firm ground as shown in Fig. 7b,c. The piles needed to build levees-walkways in soft, muddy sediments, would not have been necessary on the firm sandy silt sediments overlying the “caranto” paleosol. In other words, the construction of the road was probably adapted to the topography, geomorphology, and ground conditions.

In this regard, Xeidakis and Varagouli^96^ documented the different construction techniques for the northern Greek part of the Roman *Via Egnatia* crossing the Balkan Peninsula. They observed that the foundation conditions determined the thickness and layering of the pavement: “In stable, rocky ground, the pavement consisted of only one layer of well-fitted cobble stones; whereas, in soft and unstable ground the soft soil was excavated and replaced by several layers of cobbles, gravels and rubbles held together with compacted sandy soil or lime mortar. [...] The thickness of the pavement varied from 25 cm to more than 150 cm”.

Matteazzi drew similar conclusions in his review study about the technical construction choices of Roman roads in northern Italy^97^. The foundation of the roads he investigated presented only one or two layers of material such as gravel, sand or clay, sometimes enriched by fragments of stones or bricks. This modest structure is quite different from the one testified by the archaeological studies in central Italy^98^. Matteazzi concluded as well that this is related to the geological substrate of the Po Plain, where banks of consolidated clay guaranteed the solidity of the road structure without the need of complex foundations, that were instead necessary for humid swampy areas.

Finally, we cannot even exclude that the TC road was used as tow paths for the journeys in the Venice Lagoon in a complex infrastructural system. In fact, if we consider also the southern part of the study area, the presence of the structure a (Fig. 2) to the south-west of the Treporti channel could possibly represent a large harbour structure located at the southern edge of the TC paleobeach ridge with a long wall and a central tower probably located in correspondence to a southern entrance to the lagoon^40,68^. This structure could correspond to the sites 113.2a identified by Canal (Fig. 6), who did not describe it but indicated it in the map with the symbol of a Roman wall. Its dimension (15 × 10–12 m, Profiles I–II and II–IV, respectively, in Fig. 2) are comparable to those of the building found in the S. Felice Channel interpreted as a possible defensive tower or a lighthouse^11,23–25^ (Fig. 6, 112). This structure, however, lay much deeper (8–9 m) than the current depth of the TC road (about 4–5 m). Possibly, the TC road was built on a supratidal zone, whereas the structure could have been a harbour structure built in the intertidal zone in correspondence of an ancient inlet. Thus, the structure could have been more exposed to storm-wave pounding causing its subsidence in the sand. Indeed, different rates of natural and anthropogenic subsidence have been observed in the Venice Lagoon with recent higher vertical displacements close to the inlets^43,73^. Also, the comparative analysis of historical hydrographic maps show that strong erosive processes have occurred in the last century close to the Lido inlet^69^: the southern part of the investigated area (depicted in Fig. 2) deepened by 3 to 6 m between 1897 to 2003, i.e., after the construction of the jetties in the Lido inlet.

Moreover, the structure could have been originally built, at least partly, under water like harbour installations such as breakwaters, jetties, docks^17^. However, further archaeological investigation is needed to determine the functional elevation of the structure, namely the relationship between the emerged part of the archaeological remains compared to past and present mean sea level^16,99^.

### A comparison with the Lagoons of Grado and Marano

An overview to the maritime landscape of Aquileia represents a valid comparison with the Venice Lagoon case study with a focus on the Grado and Marano Lagoons, that have stringent affinities with the basin of *Altinum*. Recent studies about the Grado Lagoon have demonstrated the wide extension of the land in the Roman age in the area now submerged^100^. From the late Republic phase to the early Empire there was a big flat area with infrastructures linked both to the rivers and to the maritime trades: there were residential and harbour settlements, as well as places used for cult and funerary purposes (Figure A2 in Supplementary Material). Among the archaeological sites, there were some storages (at Marina di Macia and in the seabed of Groto/Island of Pampagnola), docks, moorings, private buildings (at Le Cove), that contributed to offer a first stop and refreshment to people who were sailing for long periods. They seem similar to the buildings found in the northern Venice Lagoon between the Treporti and Lio Piccolo areas.

These ancient sites in the Grado Lagoon now submerged were served both by waterways (channels) and roads. In particular, a ‘speaking’ site, i.e., the place Pietre/Piere (Stones) of Sant’Agata-San Gottardo (S. Gottardo in Figure A1 in Supplementary Material), is located in the sea at 600 m from the current dam of Grado and at 3 m of depth^101^. The site is characterised by an alignment of stones that has been interpreted as a road or a quay. De Grassi suggested the importance of the several funeral artefacts discovered through the years between the Grado beach and the ‘Ruins of S. Gottardo’.

A second, important example of a submerged road seen in the Grado Lagoon connected the sea to Aquileia: it is a long road close to the modern sites of Morsano and Grotto, near the Groto-Pampagnola Island, the Gorgo Island-Villa Nova, and the area of Morsano (Figure A2 in Supplementary Material, red dashed line). Here, a big structure constituted by an alignment of walls for over 40 m has been interpreted as part of the harbour of the Aquileia Lagoon, perhaps with a wood pier^100^. Overall, the Grado finds offer a strong comparison with the Treporti Channel in the Venice Lagoon, showing a clear example of routes that guaranteed the land passage in an ancient lagoon of the north Adriatic Sea.

The Marano Lagoon presents further examples to be compared with the Venice Lagoon (Figure A2 in Supplementary Material). The lagoon formed about 7500 years ago and in the Roman age it presented some emerged areas, that were progressively submerged^102^; only a few places have been preserved dry thanks to a water-scooping system realized at the beginnings of the XX century^103^. In this case, as well, the submersion of the archaeological lands depended on multiple factors: the natural subsidence, climate change, as well as the anthropogenic action, such as, for instance, the creation of wells and the consequent exploitation of groundwater. The Marano basin is much shallower than the Venice Lagoon with an average depth of about 70 cm: for this reason, in this area the archaeological diggings have been convenient, and the consistency of the archaeological remains has been easier to document.

The Marano Lagoon was located between the sea mouths of the *Anaxum-Stella, Alsa-Aussa, Corno* rivers and it was useful for all the network of trades linked to Aquileia^104,105^. As in the case of *Altinum*, three types of routes could have connected Aquileia: (i) the land route, thanks to the consular roads in the Augustan *Venetia et Histria Regio* created by Rome starting from the constitution of Aquileia in 181 BC^106^; (ii) the sea route, in the Adriatic courses sailed from the Mycenaean and Pre-Roman age^107^; (iii) the internal waters route, strengthened by the artificial channels that defended the sailors from bad weather and piracy^22,76^. The most famous artificial channel that linked the Marano Lagoon to Aquileia was the Canale Anfora^13,108–110^: as in the case of the Sioncello Channel in the *Altinum* Lagoon, it represented a navigable route integrated into the local hydrography and it was coherently oriented along the Aquileia centuriation during the Flavian Empire.

### Which road system in the ancient Venice Lagoon?

The different archaeological contexts mentioned above offer comparisons with the case study of the Treporti Channel in the Venice Lagoon presenting similar types of settlements. The road system in the Aquileia basin is well documented by the submerged route that linked the Grado Lagoon to Aquileia. In the case of the Venice Lagoon the road system is still to be defined because of two elements. On the one hand, the lack of wide-ranging underwater stratigraphic excavations, on the other hand, a widespread reticence also in the academic field to recognise an ancient landscape largely emerged and therefore crossed and inhabited.

**Figure 8:**
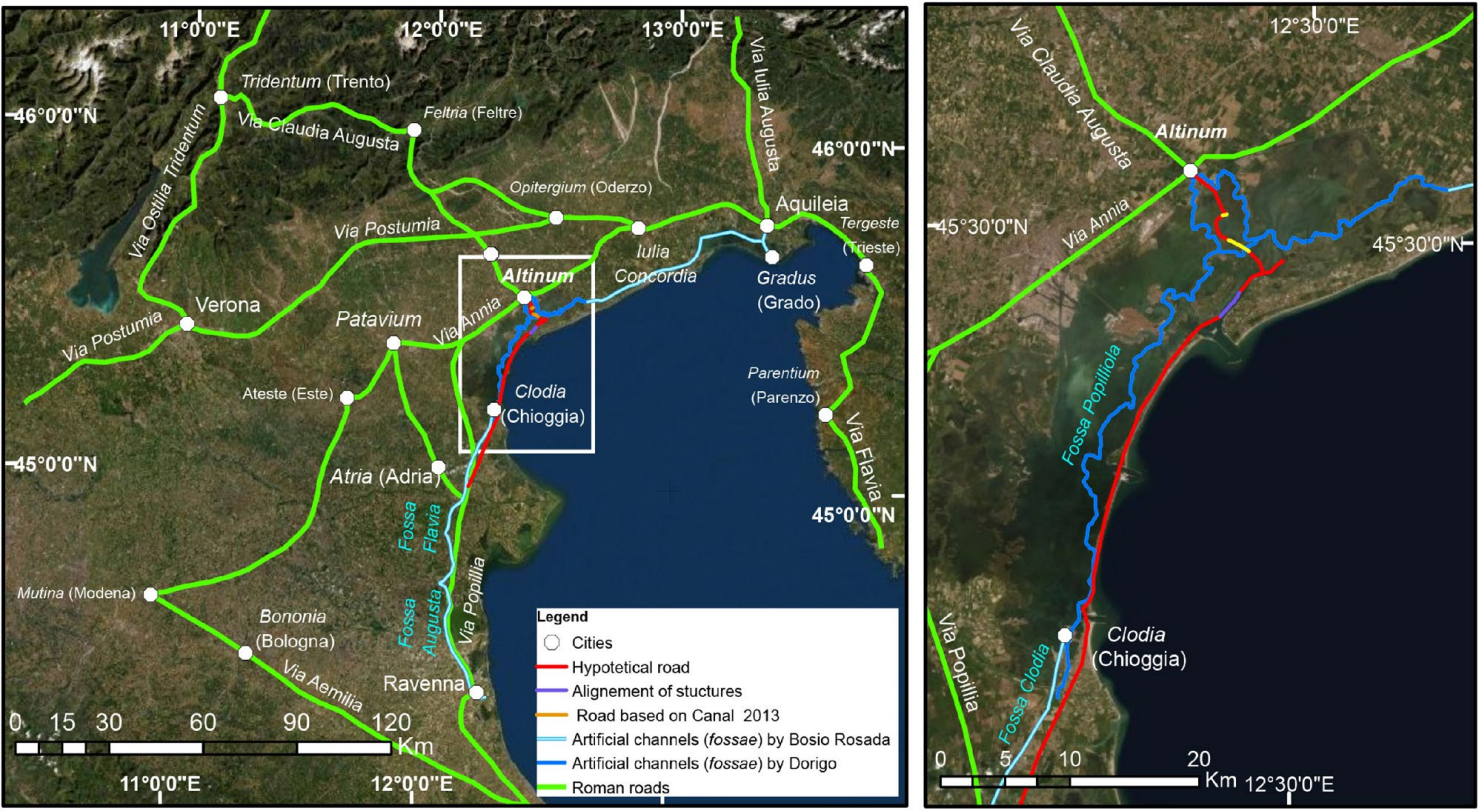
(*Left*) Roman road and waterways network in the Augustan *Venetia et Histria Regio* in the North-East Italy. (*Right*) zoom on the Venice Lagoon showing the *fossa Popilliola* in blue (after Dorigo^22^) and the hypothetical path of the Roman road along the littoral in red, with the TC road segment in purple and the segment reconstructed from Canal^11^ (Fig. 6, 123), in yellow. Satellite image source: Esri DigitalGlobe, GeoEye, i-cubed, USDA, USGS, AEX, Getmapping, Aerogrid, IGN, IGP, swisstopo, and the GIS User Community, https://services.arcgisonline.com/ArcGIS/rest/services/World_Imagery/MapServer.

The elements highlighted by the multibeam survey in the Treporti Channel connected to the geo-archaeological underwater findings, allow us to start outlining the ancient viability in the maritime area of the *Venetia*, even if only in a small section (Fig. 8). This viability was not limited to scattered paths, but it was linked to the wide road Roman network that offered convenient passages in all the Veneto territory, both in its continental extension and in its lagoon spaces. In fact, as in all the ancient Po valley, the road system of the Veneto Region included different routes (Fig. 8). There were the consular roads built between the II century BC and the I century AD (*via Postumia, Annia, Popillia, Claudia Augusta*^106^, from which minor paths allowed one to reach secondary sites (in green in Fig. 8). There were the river routes, that guaranteed the transports of goods for long distances thanks to complex infrastructures. But there were also the famous *fossae per transversum*, the artificial channels dug in the ancient lagoons between the mouths of the rivers, that extended parallel of the littoral coasts and that allowed an internal save navigation, avoiding the open sea^25,111,112^ (in light blue and blue in Fig. 8).

It is possible then to reconsider the TC road within the wider perspective of the Roman viability in the ancient Veneto Region: the submerged road represented the last section of a vast route system both on land and water starting from the south, in correspondence with the contemporary city of Chioggia. It further continued northward crossing the modern Malamocco littoral, where the harbour of the ancient river *Meduacus* has been pinpointed (the sea harbour of *Patavium*-Padova; the toponym derives from the ancient name of the river *Metamaukos/Meduacus*, that in Antiquity flowed here^113^); finally, it ended in the Northern Venice Lagoon and possibly continued until the Aquileia basin. This route system, protected from coastal storms and piracy, provided both land paths, as the case of the TC road, and waterways allowing the boats to pass from one end of the northern Adriatic Sea to the other. These channels had such a strategic role—political, economic, and social—, that Plinius the Older described in a famous page of his *Naturalis Historia* (Plin. nat. III, 119–121).

The enhancement of the land and water viability, already started by the Etruscans, has to be referred to the Augustan project to unify the Italian peninsula: the *tota Italia* mentioned by the *Princeps* himself in his *Res Gestae* (*Res Gestae*, 25, 1–2^114^), after the conquer of the Alps and the submission of all the people of the Cisalpine territories. The Augustan project provided a new management and a new re-organization of the infrastructural system of the north-eastern Adriatic area, starting from the constitution of two harbours at Ravenna: the harbour of *Classis*, for the allocation of part of the imperial fleet, and the harbour of Ravenna, dedicated to trade activities. From here, the channels already made during the Pre-Roman phase were renovated. Unifying different river mouths (of the Po, Adige, Brenta, Sile-Piave rivers), the internal lagoon routes included the *fossa Augusta*, that linked Ravenna to Spina, the *fossa Flavia* between Spina and Adria, the *fossa Clodia* from Adria to Chioggia, finally a channel, mentioned in Medieval sources, called *fossa Popilliola*, that allowed the passage from Chioggia to *Altinum* and beyond^76^ (Fig. 8). This artificial channel coincided with an itinerary that started from the consular road *Via Popillia* (made by *P. Pupillius Lenas* in 132 BC) from Rimini to *Altinum* (an important example of a Pre-Roman docking that become a *Roman mansio* in front of the lagoon is attested at San Basilio, Ariano Polesine-Adria^115^). Close to the *mansio Maio Meduaco* (today Sambruson, near Dolo), the *Via Popillia* was unified to the consular road *Via Annia* until Aquileia, with multiple ‘service stations’ along the way. It was an amazing viability project, providing the choice among different routes by land or by water, diffusely connected with facilities for breaks or stops, for water provisions, and for selling activities. These journeys in the north-east Adriatic area may have been integrated in multiple itineraries over the Alps passing through the *via Claudia Augusta*: this famous consular road started by land in *Altinum* but, as proposed in a recent and deepened study^116^, it could begin in a maritime path from the *fossa Augusta*, to pass through the Venice Lagoon and to arrive in *Altinum*. From here it continued by land, until *Tridentum* and the Danube area.

## Conclusions

This multidisciplinary study documented the presence of an about 1200 m long segment of a submerged Roman road (called for simplicity TC road) on an ancient beach ridge now submerged in the Northern Venice Lagoon. The high resolution underwater acoustic data collected was interpreted in light of geological data, archaeological excavations and comparisons with similar environments and archaeological contexts. In the attempt to preserve this precious underwater cultural heritage, we presented a reconstructive hypothesis and a 3D digitization of the the paleobeach ridge and of the TC road, that are currently endangered by erosion and subsidence processes.

The submerged road represents, probably, one of the last route segments in the maritime landscape of *Altinum*, within a wider network of roads in the *Venetia et Histria Regio*. Its contiguity with other structures and infrastructures, such as for instance defensive towers, levees-walkways, port, and private structures, confirms the capillary permanent settlement in the *Venetorum angulus*. This area was crossed by travellers and sailors, namely the *viatores et velatores*, to which the freedwoman Aufidia Venusta wished good health in a funeral inscription found at Portomaggiore, close to Ferrara (EDR140752 = CIL 05, 02402 = Suppl It, 17, 1999, p. 150):

*Aufidiae C(ai) l(ibertae) Venust[a]e/viatores et velatores salvete/et bene valete.*

## Methods

### The high-resolution seafloor mapping

Detailed geoarchaeological observations and analyses were carried out in the Treporti channel. We studied the seafloor geomorphology of this area thanks to underwater acoustic remote sensing. During an extensive survey carried out in 2013, the high resolution multibeam data were collected with a Kongsberg EM-2040 DC dual-head system with 800 beams (400 per swath). The MBES was pole-mounted on the bow of the vessel RV Litus, a 10-m long boat with 1.5-m draft. The frequency of MBES was set to 360 kHz. A Seapath 300 system provided the position corrected with a Fugro HP differential Global Positioning System (dGPS, accurate to 0.20 m). The Kongsberg MRU5 inertial motion sensor corrected pitch, roll, heave and yaw movements (0.02° roll and pitch accuracy, 0.075° heading accuracy). A Valeport mini SVS sensor mounted close to the transducers measured continuously the sound velocity for the beam forming. Sound velocity profiles were systematically collected with an AML oceanographic Smart-X sound velocity profiler. Data were logged, displayed, and checked in real-time with the Kongsberg native data acquisition and control software SIS (Seafloor Information System). The processing of the raw data was done with the software CARIS HIPS and SIPS^117^ (v.9) that accounted for sound velocity variations, tides and basic quality controls. A set of 93 virtual tidal stations evenly distributed in the study area, served for tidal correction: a virtual tidal station, was used for each field sheet of CARIS created for the data collected in a single day of survey. The tidal corrections in each virtual tidal station were calculated using the water level simulated by the hydrodynamic model SHYFEM^118,119^ applied to the whole Venice Lagoon with assimilation of tide gauge observations. All the depths are referred to the local mean sea level datum ‘Punta Salute 1897’. The bathymetric grids were exported from CARIS as text files with grid resolutions ranging from 0.05 to 0.5 m. They were converted to 32-bit raster files using Global Mapper (v12) (see further details in Madricardo et al.^120^).

The morphology of the sites and their surrounding areas were studied through the analysis of Digital Terrain Models (DTM) obtained from the bathymetric grids with a resolution of 0.2 m. They were analyzed with the software ArcGIS (v10.2)^121^. The visual interpretation of the DTMs was the basic tool for detecting traces of the archaeological structures. The recognized elements were drawn and all the information concerning each structure were automatically extracted using the tools *Add feature attributes*, *Boundary * and *Focal statistics* to extract the area, perimeter, maximum width and length, the minimum, maximum and mean depth of each structure.

### Underwater archeological inspections

Underwater archaeological researches were carried out in the Treporti Channel between 1978 and 2016. In particular, the first survey was realized by Antonio e Paolo Molino, Eros Turchetto and Paolo Zanetti on behalf of the ‘Soprintendenza Archeologica del Veneto’ between July and November 1985. The report written about this survey represents the starting point of our archaeological study. During the survey, the underwater operations were particularly difficult due to strong currents and intense boat traffic. The investigation had the main objectives of determining exactly the location of the site and the extension of the archaeological evidence, characterizing the materials and the substrate composition of the area.

To identify the site, divers carried out parallel transects putting in the middle of the area a buoy as a reference to extract the geographical coordinates in the moment of slack water in the tidal cycle when the current was practically absent. The coordinates of the buoy where then calculated with a sextant measuring the angle with two known points on the mainland and other reference points were measured at the site extremes. The morphology and the different depths of the site were then measured by divers with the help of a metered pile and a level. The main direction of the site was determined with the help of a compass.

Four sediment cores were extracted at the same depth of 4.5 m under the lithic layer in order to characterize the site substrate. The cores were collected with a PVC tube with a diameter of 4 cm and a length of 1 m. The recovered cores were then analyzed in the lab to extract sedimentological and micropalaeontological records. Two lithic elements were collected, and thin sections were extracted for the minero-petrographic analysis. The operators used a water dredge working as a suction device to remove sediment from the seabed and to study the structure. In July 2020, further video documentation was acquired by the divers of the Polizia di Stato in the Treporti Channel by mean of a camera Canon EOS 60D following a transect in the direction NE-SW in the Treporti Channel in correspondence with the structure 3 identified with the MBES (Fig. 2).

### The 3D digitization

In a project of digitization of a cultural asset, it is possible to distinguish several consecutive and sequential phases. The metric and configuration survey is entrusted with the first phase of reading, and it currently uses the most modern technologies applied to this field—in this case the high-resolution MBES. The next phase consists in the elaboration and optimisation of the data obtained with reverse engineering techniques through digital modelling software for the creation of clones characterised by geometric-mathematical specifications^122^. The final phase deals with digital systems of cataloguing and diffusion of the asset through innovative techniques of immersive media: video mapping, apps for mobile devices, augmented reality, immersive $360 ^\circ$ videos, etc., which nowadays guarantee an easily usable and involving diffusion of the conceptual contents typical of an artefact under analysis^123^.

If the ultimate purpose of the entire work program is the creation of the digital clone, then it is perhaps useful to underline that the model, both digital and physical, must immediately abandon the ambition to structure itself as a ‘faithful copy’ of the real data. This model must be structured as a semantic one, with a strong critical and interpretative character, capable of storing and communicating not only the visible form (or the form reconstructed based on archive documentation) but also, and above all, the most intimate and profound meaning of that same form. It is precisely the meaning of the form that gives that asset the adjective ‘cultural’, and the model cannot and must not ignore that meaning. The reconstructive hypothesis of the coast of the Treporti area in Roman Times (Fig. 8), therefore, is obtained through a reverse modelling process in order to deduce the object shape representation from the digital acquisition of the physical model. In this specific case, the three-dimensional survey data collected through the MBES were integrated with a series of heterogeneous data obtained mostly from archive and geo-archaeological data in order to propose a possible visual reconstruction of the places that are now ‘submerged’, both physically and in the historic memory.

The first step concerned the transformation of the discrete three-dimensional model (point cloud) into a polygonal mesh model. The point cloud is a set of points characterised by their position in an XYZ coordinate system and by intensity values and/or RGB values, in case of a 3D laser scanner or photogrammetric survey. In this specific case, the values provided by the bathymetric survey were only of metric nature, that is XYZ. For the purposes of the three-dimensional modelling, the point cloud constitutes the metric starting point, a dotted model that cannot be modified. On the other hand, the polygonal mesh is a polyhedral surface characterised by flat facets, usually triangular or quadrangular. These types of models give an appreciation of the geometric trend of the surveyed object’s surface. Mesh models, because of their characteristics, are the most commonly used to create the efficient topological models of surfaces with casual variation—such as soils and depths—, the so-called Digital Terrain Model. The meshing process consisted in the realization of a triangular Triangular Irregular Network (TIN) mesh using the reverse engineering software Geomagic Design X (https://it.3dsystems.com/software/geomagic-design-x). The triangles of the mesh have sides that connect the set of the dots measured on the object, with the geometric characteristic of uniquely identifying a flat surface in space. The result is therefore a polyhedral surface composed of triangular elements.

The archaeological evidence was subsequently identified on the mesh surface and became the remarkable points on which cross sections of the model were extracted. These sections were the starting point for the construction of the new digital model—the hypothetical reconstruction one. In this phase, which was developed in the digital environment of Rhinoceros (https://www.rhino3d.com/it/), NURBS (Non-uniform rational B-spline) digital modeler, the data of the survey were integrated by the geo-archaeological and archive data to allow the reconstruction of the curves supporting the model. Then, the new section curves were interpolated and generated a continuous mathematical surface. The geometry represented by NURBS modelers is mathematical and it can represent any type of surface accurately and continuously. The model was then rendered with the software Cinema4D (https://www.maxon.net/en/products/cinema-4d/overview/) and V-ray (https://www.chaosgroup.com/). The rendering engines can simulate and graphically replicate a series of physical phenomena like shadows, reflection, caustics, subsurface, scattering and ambient occlusion, to cite a few of them. The mathematical model created by NURBS was imported in Cinema 4D, where each surface was associated with a different material to simulate the reality (water, soil, stone, etc.), adding also the solar light, i.e. the so called “physical sky”. After the setting of these parameters, the cameras were positioned to obtain different perspectives and then the rendering procedure was launched to obtain the desired views (Fig. 7).

## Supplementary Information

Supplementary Information.
