# Supplementary material for: New evidence of a Roman road in the Venice Lagoon (Italy) based on high resolution seafloor reconstruction
Source: Sci Rep. 2021 Jul 22;11:13985. doi: 10.1038/s41598-021-92939-w (PMC8298408; doi:10.1038/s41598-021-92939-w)
Supplement: Supplementary file 1 — Supplementary Information. [file 41598_2021_92939_MOESM1_ESM.pdf]

New evidence of a Roman road in the Venice Lagoon (Italy) based on high resolution seafloor reconstruction

Fantina Madricardo[1,*], **Maddalena Bassani**[2], **Giuseppe D'Acunto**[2], **Antonio Calandriello**[2], **and Federica Foglini**[3]

[1]CNR-National Research Council, ISMAR-Marine Sciences Institute in Venice, Castello 2737/f, 30122 Venice, Italy
[2]Università Iuav di Venezia, Santa Croce 191 Tolentini 30135 Venice, Italy
[3]CNR-National Research Council, ISMAR-Marine Sciences Institute in Bologna Via Gobetti, 101, 40129 Bologna, Italy
[*]corresponding authors: fantina.madricardo@ismar.cnr.it

ABSTRACT

This study provides new evidence of the presence of an ancient Roman road in correspondence to a paleobeach ridge now submerged in the Venice Lagoon (Italy). New high resolution underwater seafloor data shed new light on the significance of the Roman remains in the lagoon. The interpretation of the data through archive and geo-archaeological research allowed a three-dimensional architectural reconstruction of the Roman road. The presence of the ancient Roman road confirms the hypothesis of a stable system of Roman settlements in the Venice Lagoon. The study highlights the significance of this road in the broader context of the Roman transport system, demonstrating once more the Roman ability to adapt and to handle complex dynamic environments that were often radically different from today.

A Supplementary material

Point number	*Lat.*	*Long.*	*Lab code*	*RSL (m)*	*RSL uncert. + (m)*	*RSL uncert. - (m)*	*Age BP (ys)*	*Age uncert. (ys)*	*Material*	*Age CE (ys)*	*Error (ys)*	*Reference*
1	45.4	12.25	ROME-1205	-1.1	0.5	0.5	592	66	Bulk material	1359	66	Serandrei-Barbero et al., 2006
2	45.35	12.32	OZG-805	-0.7	0.6	0.6	783	110	Bulk material	1168	110	Serandrei-Barbero et al., 2006
3	45.47	12.41	OxA-10717	-1.6	1.2	1.2	812	102	Plant remains	1138	102	McClennen and Housley, 2006
4	45.48	12.33	OZ-G311	-0.9	0.6	0.6	930	126	Shell	1021	126	Serandrei-Barbero et al., 2006
5	45.35	12.32	OZG-332	-0.4	0.4	1.0	1261	199	Shell	689	199	Serandrei-Barbero et al., 2006
6	45.47	12.41	OxA-10722	-0.7	0.7	1.2	1463	84	Plant remains	487	84	McClennen and Housley, 2006
7	**45.49**	**12.35**	**OZ-G317**	**-0.3**	**0.3**	**1.1**	**1532**	**211**	**Shell**	**419**	**211**	**Serandrei-Barbero et al., 2006**
8	45.36	12.25	OZG-320	-1.1	0.6	0.6	1680	212	Shell	271	212	Serandrei-Barbero et al., 2006
9	45.47	12.41	OxA-6784	-0.6	0.6	0.7	1696	127	Wood	254	127	Zoppi et al., 2001
10	45.47	12.41	OZF-487	-1.4	1.2	1.2	1740	126	Plant remains	210	126	McClennen and Housley, 2006
11	45.35	12.32	OZG-333	-0.7	0.7	1.0	1806	217	Shell	144	217	Serandrei-Barbero et al., 2006
12	45.47	12.41	OxA-8629	-0.9	0.7	0.7	1824	100	Leaf	126	100	Zoppi et al., 2001
13	45.47	12.41	OZ-E696	-1.0	0.7	0.7	2047	198	Foraminifera	-97	198	Zoppi et al., 2001
14	45.47	12.29	LTL-1631A	-1.4	0.6	0.6	2503	191	Peat	-553	191	Madricardo and Donnici, 2015
15	**45.35**	**12.32**	**OZG-322**	**-1.4**	**0.7**	**0.7**	**2584**	**233**	**Foraminifera**	**-634**	**233**	**Serandrei-Barbero et al., 2006**
16	45.54	12.44	CARG-12	-0.9	0.9	1.2	3093	122	-	-1143	122	Tosi et al., 2007
17	45.47	12.41	OZ-E697	-2.2	0.7	0.7	3561	187	Shell	-1611	187	Zoppi et al., 2001
18	45.47	12.41	OZ-E698	-2.2	0.7	0.7	3635	188	Foraminifera	-1685	188	Zoppi et al., 2001
19	45.47	12.41	OZF-484	-2.0	1.2	1.2	4075	342	Plant remains	-2125	342	McClennen and Housley, 2006
20	45.47	12.41	OZ-E699	-1.8	0.7	0.7	4189	207	Foraminifera	-2239	207	Zoppi et al., 2001
21	45.47	12.41	OxA-1076	-1.0	1.0	1.2	4250	157	Plant remains	-2300	157	McClennen and Housley, 2006
22	**45.48**	**12.41**	**GX-26939**	**-4.3**	**0.8**	**0.8**	**4673**	**147**	**Peat**	**-2723**	**147**	**Lezziero et al., 2002**
23	**45.43**	**12.45**	**OZF-080**	**-3.0**	**0.6**	**0.6**	**5556**	**237**	**Shell**	**-3606**	**237**	**Donnici et al., 2012**

Table 1. RSL index points for the Venice Lagoon area extracted from the RSL database of the western Mediterranean coast included in Vacchi et al.[1], where all the References can be found. The basal points are in bold.

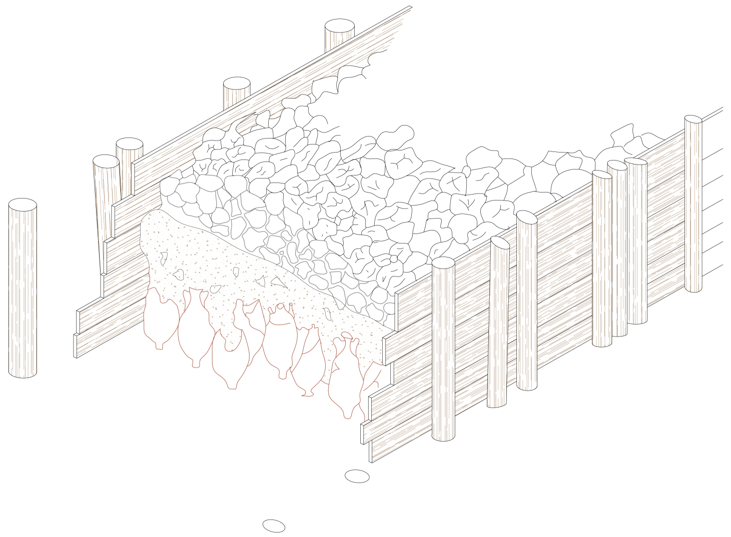

Figure A.1. Structure of the submerged levee-walkway discovered in the S. Felice Channel made of a wooden caging, amphorae, infill materials and stone blocks to cover the top of the way (modified from Fozzati and Toniolo[2]).

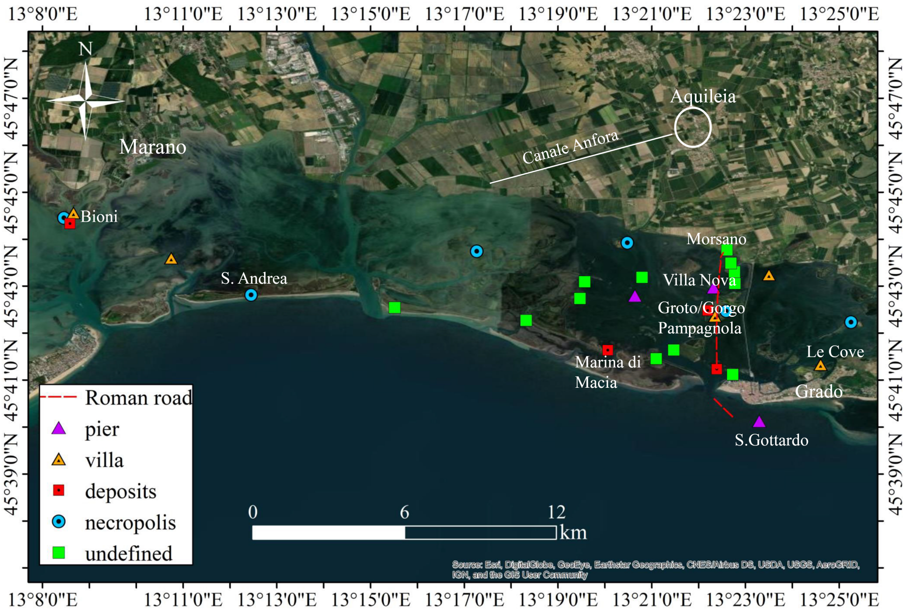

Figure A.2. Map of the Grado and Marano Lagoons with the most important archaeological findings (modified from[3,4]. Satellite image source: Esri DigitalGlobe, GeoEye, i-cubed, USDA,USGS,AEX,Getmapping, Aerogrid, IGN, IGP, swisstopo, and the GIS User Community,
https://services.arcgisonline.com/ArcGIS/rest/services/World_Imagery/MapServer

- The MBES data are available from the repository: Madricardo, F., Foglini F and. Trincardi F. Marine Geosciences Data System. http://dx.doi.org/10.1594/IEDA/323605
- A short documentary related to this study with the title "La Venezia Romana" realized by DocLab Srl for National Geo-

graphic can be found at the link: https://www.nationalgeographic.it/video/tv/la-venezia-romana/

References

1. Vacchi, M. *et al.* Multiproxy assessment of holocene relative sea-level changes in the western mediterranean: Sea-level variability and improvements in the definition of the isostatic signal. *Earth-Science Rev.* **155**, 172–197 (2016).
2. Fozzati, L. & Toniolo, A. Argini-strade nella laguna di Venezia. *Bonifiche e drenaggi con anfore epoca romana: aspetti tecnici e topografici* 197–208 (1998).
3. Gaddi, D. Approdi nella laguna di Grado. *Antichità Altoadriatiche* (2001).
4. Auriemma, R. *et al.* Alle porte del mare. La laguna di Marano in Età romana. *Antichità Altoadriatiche* (2013).
